# The application of health literacy measurement tools (collective or individual domains) in assessing chronic disease management: a systematic review protocol

**DOI:** 10.1186/s13643-016-0267-8

**Published:** 2016-06-07

**Authors:** Jessica Shum, Iraj Poureslami, Mary M. Doyle-Waters, J. Mark FitzGerald

**Affiliations:** Vancouver General Hospital - Research Pavilion, 7th floor, 828 West 10th Avenue, Vancouver, BC V5Z 1M9 Canada; The Lung Centre, Gordon and Leslie Diamond Health Care Centre, 7th floor, 2775 Laurel Street, Vancouver, BC V5Z 1M9 Canada

**Keywords:** Health literacy, Asthma, Chronic obstructive pulmonary disease, Measurement tools, Domains, Systematic review

## Abstract

**Background:**

The term “health literacy” (HL) was first coined in 1974, and its most common definition is currently defined as a person’s ability to access, understand, evaluate, communicate, and use health information to make decisions for one’s health. The previous systematic reviews assessing the effect of existing HL measurement tools on health outcomes have simply searched for the term “health literacy” only to identify measures instead of incorporating either one or more of the five domains in their search. Furthermore, as the domain “use” is fairly new, few studies have actually assessed this domain. In this protocol, we propose to identify and assess HL measures that applied the mentioned five domains either collectively or individually in assessing chronic disease management, in particular for asthma and chronic obstructive pulmonary disease (COPD). The ultimate goal is to provide recommendations towards the development and validation of a patient-centric HL measurement tool for the two diseases.

**Methods/design:**

A comprehensive, electronic search will be conducted to identify potential studies dating from 1974 to 2016 from databases such as Embase, MEDLINE, CINAHL, Cochrane Central Register of Controlled Trials, Web of Science, ERIC, PsycINFO, and HAPI. Database searches will be complemented with grey literature. Two independent reviewers will perform tool selection, study selection, data extraction, and quality assessment using pre-designed study forms. Any disagreement will be resolved through discussion or a third reviewer. Only studies that have developed or validated HL measurement tools (including one or more of the five domains mentioned above) among asthma and COPD patients will be included. Information collected from the studies will include instrument details such as versions, purpose, underlying constructs, administration, mapping of items onto the five domains, internal structure, scoring, response processes, standard error of measurement (SEM), correlation with other variables, clinically important difference, and item response theory (IRT)-based analyses. The identified strengths and weaknesses as well as reliability, validity, responsiveness, and interpretability of the tools from the validation studies will also be assessed using the COSMIN checklist. A synthesis will be presented for all tools in relation to asthma and COPD management.

**Discussion:**

This systematic review will be one of several key contributions central to a global evidence-based strategy funded by the Canadian Institutes of Health Research (CIHR) for measuring HL in patients with asthma and COPD, highlighting the gaps and inconsistencies of domains between existing tools. The knowledge generated from this review will provide the team information on (1) the five-domain model and cross domains, (2) underlying constructs, (3) tool length, (4) time for completion, (5) reading level, and (6) format for development of the proposed tool. Other aspects of the published validation studies such as reliability coefficients, SEM, correlations with other variables, clinically important difference, and IRT-based analyses will be important for comparison purposes when testing, interpreting, and validating the developed tool.

**Systematic review registration:**

PROSPERO CRD42016037532

**Electronic supplementary material:**

The online version of this article (doi:10.1186/s13643-016-0267-8) contains supplementary material, which is available to authorized users.

## Background

The most common definition for health literacy (HL) has been defined as “the degree to which individuals have the capacity to obtain, process, and understand basic health information and services needed to make appropriate health decisions” [1, 2]. In 2008, the Canadian Expert Panel on Health Literacy (CEPHL) [3] developed a model of HL which included four main domains and defined HL as a person’s ability to (1) access, (2) understand, (3) evaluate, and (4) communicate health information in order to make sound health decisions. A year later, the Calgary Charter on Health Literacy (CCHL) international conference [4] added a fifth domain of the “using” of health information as a critical component to the existing model. The importance of HL for each of these domains has been well established *individually* [5, 6], and the CCHL’s suggested “five-domain model” has been endorsed and approved by different HL researchers and experts [7, 8]. However, despite this being the case, the existing measures of HL still only focus on one or two aspects of HL, such as word comprehension or reading ability [6, 9]. Ideally, there needs to be a single comprehensive measure assessing all aspects of HL as well as health numeracy to identify the specific gaps or areas of weaknesses between each of the domains [8, 10].

Research on HL has grown tremendously in the past two decades, but the measurement of HL is still at a preliminary stage from a methodological point of view, however, mainly due to disagreement regarding the definition of HL among researchers and clinicians. Furthermore, limitations with existing tools prevent us from effectively assessing and measuring HL [5–10]. More specifically, the existing HL tools do not adequately capture the data necessary to understand how HL, as a complex concept, is a determinant of health outcomes [8, 11] and which domains play a crucial role in this regard. Therefore, the existing tools are thus inadequate in identifying the specific areas of improvement needed within the domains [5, 6, 11, 12]. The limited scope of existing measurement tools also prohibits researchers and practitioners from identifying which mechanism(s) and approaches are needed to resolve the deficit (e.g. skill improvement, knowledge enhancement, empowerment, behavioural modification). Instrument reliability was often low, and most importantly, none of the current available tools measure how patients apply or “use” health information to make informed decisions to manage their health condition [13, 14]. The three most widely used measures of HL [2, 8, 15] are the Newest Vital Sign (NVS) [16, 17], the Rapid Estimate of Adult Literacy in Medicine (REALM) [18], and the Test of Functional Health Literacy in Adults (TOFHLA) [19]. These tools largely measure reading ability (verbal understanding), print literacy, or numeracy [20]. This highlights the challenge of differentiating between health literacy and basic literacy. Additionally, these instruments, as well as many other HL tools, fail to address other aspects of the five-domain HL model. For instance, NVS and REALM focus mainly on assessing an individual’s comprehension, pronunciation of health-related terms, and numeracy, yet health literacy is a broad concept of an individual’s skills, abilities, and knowledge. TOFHLA focuses on reading and verbal literacy, which limits our understanding of HL deficiency among studied participants.

As a determinant of health [3, 19], HL affects a person’s ability to access and use health care, to interact with care providers and health professionals, and to make sound decisions for their own health. HL measures have generally followed this model, focusing on measuring an individual’s capabilities rather than actual functional HL skills and without reference to any interaction he or she may have with health information or the health care system. Practitioners and HL researchers have debated the practicality of existing measurement tools for accurately assessing HL in middle-aged and older adults [3, 4, 7, 21–23].

In 2011, Berkman et al. [24] updated the results of their 2004 systematic review [25] on HL and health outcomes and identified that low literacy was associated with severe adverse health outcomes and greater use of health care services such as increased hospitalizations, greater use of emergency care, and worse ability to interpret prescription labels and health messages. On the positive side, Berkman et al. also concluded that improved HL was associated with better health outcomes. Sørensen et al. [26] conducted a systematic review in 2012 classifying the definitions and conceptual frameworks of HL and developed a conceptual model containing 12 dimensions referring to knowledge and motivation for accessing, understanding, and applying health information within the health care and health promotion setting.

Few reviews have focused on the deficiencies of health literacy measurement tools. For instance, in 2011, a critical appraisal of HL was done by Jordan et al. [6] evaluating 19 instruments and they found wide variations of constructs and content across instruments, and none appeared to fully measure a person’s ability to seek, understand, and use health information. Content was mainly focused on reading comprehension and numeracy, scoring categories were poorly defined, and very few questionnaires had been assessed for reliability. Collins et al. [27] systematically reviewed 11 different HL instruments used for mobile health information and technology screening and evaluation tools in 2012 and also found that there was a lack of consistency in the types of screening questions proposed. This demonstrates that current health literacy screening tools provide varying benefits depending on the context of their use. Most recently, Kiechle et al. [28] conducted a systematic review on performance-based versus self-reported measures of health literacy and numeracy. A total of ten studies were included in the final assessment. They concluded with mixed results; the performance-based measures often target skills such as reading comprehension, word recognition, and basic facility with numbers whereas self-reported measures generally assess a patient’s perceived ability to perform a task, also assessing confidence and social resources and skills. More studies are needed to directly compare such tools. Kwan et al. [29] conducted a comprehensive literature review to identify the gaps in existing health literacy measurement tools. They compared the properties of each tool and clearly identified their strengths and limitations aiming to develop the content of a measurement tool for older adults in the Canadian health context. A conceptual framework for health literacy and an English version of the tool was developed and tested among an older population group. Although this is a promising accomplishment, it was not disease specific and their tool was not validated by the same team or other researchers. To our knowledge, no work has been done on evaluating HL, its measurement, and effect on health outcomes as related to asthma and chronic obstructive pulmonary disease (COPD) management; therefore, we propose to conduct this systematic review justified by three main points:

1. Previous systematic reviews searched for the term “health literacy measurement” only and did not consider studies that measured the individual domains of health literacy.
2. The domain “use” was not included in the reviewed articles of previous systematic reviews.
3. None of the previous work exclusively focuses on the functional HL skills among adult asthma and COPD patients.

### Study aims and objectives

The aim of this systematic review will be to identify whether any of the existing measurement tools aimed to assess chronic disease management among asthma and COPD patients have either collectively or individually incorporated the five domains of HL (access, understand, evaluate, communicate, and use) as suggested by the CCHL and endorsed by different researchers and studies and to assess the validity and reliability of these tools. This data will help us to identify the gaps (which HL domains are not being measured) in existing HL measurement tools and will be augmented with the information collected from patient-engaged focus groups and key-stakeholder interviews ultimately informing the research team with information to develop a comprehensive HL measurement tool capturing the five-domain model and reliably assessing how one’s HL skills may impact health outcomes. This project to develop and validate a new HL instrument has been funded by the Canadian Institutes of Health Research (CIHR) and is being initiated across Canada in a multicentre study.

With these aims in mind, the following research question and subcomponents have been designed to lead the team in generating relevant information from the review:

- Considering measurement instruments that assess one or more of the five core domains of health literacy (access, understand, evaluate, communicate, and use) specific to asthma and COPD patients, how do the items in the instruments map onto the five domains?
  - Which of the five mentioned domains are the least assessed or not included at all in existing tools?
  - Of the studies that exist on the validation of the measures, what is the evidence of reliability, validity, responsiveness, interpretability, standard error of measurement (SEM), correlations with other variables, clinically important difference, and item response theory (IRT)-based analyses of the tools?

Our systematic review was designed using the Preferred Reporting Items for Systematic Reviews and Meta-Analyses (PRISMA) guidelines [30]. A PRISMA-P checklist is available as an Additional file 1 to this protocol.

### Study inclusion criteria

HL was first discussed in the context of literacy and health [3, 5], but as the concept evolved [31–35], more definitions were suggested by different health organizations [36]. Table 1 includes the definitions for each of the five domains. Example questions for the domains can be found in Additional file 2. Eligible studies will focus on asthma or COPD patients as diagnosed by a physician or respiratory therapist in which a HL instrument pertains to one or more of the HL domains as defined below. The study population must be adult asthma or COPD patients as diagnosed by a physician or respiratory therapist. Measurement tools must assess a person’s ability in one or more of the five domains through open-ended-, closed-ended-, scenario-, passage-, puzzle-, or pictorial-type items. Tools may be self-administered or assisted through an interviewer or electronic based. The term “health literacy” was first used in 1974 during a discussion on health education as a policy issue affecting the health system; therefore, the review will include the years 1974 to 2016 [37]. Evidenced by recent systematic research, no empirical evidence of bias is reported by using only English papers and excluding papers written in non-English languages on the same topic [38].

**Table 1:** HL domains

Domain	Definition/example
Access	Being able to navigate and find health information—it is more than the availability of information and services. It is mediated by education, culture, and language, by the communication skills of professionals, by the nature of materials and messages, and by the settings in which health-related supports are provided—CEPHL [3]. o for example, I have the skills to find the health information I want.
Understand	Knowledge about a subject or situation and comprehension of the health condition and information—Cambridge Dictionaries [44]. o for example, How confident do you feel you are able to follow the instructions on the label of your inhaler?
Evaluate	To be able to determine whether information/service is applicable to self—to judge or calculate the quality, importance, truthfulness, or value of information—Cambridge Dictionaries [45]. o for example, I have the skills to judge which health information can be trusted.
Communicate	To share information with others (doctor, caregiver, family members, etc.) by speaking, writing, and body language—Cambridge Dictionaries [46]. o for example, I have the skills to describe my health concerns to others.
Use	Adapting and applying information to daily life for disease management—to take, hold, or deploy information as a means of accomplishing or achieving health outcome—Oxford Dictionaries [47]. o for example, I can use the information received from doctor/hospital to set my disease management goal.
Health numeracy	The degree to which individuals have the capacity to access, process, interpret, communicate, and act on numerical, quantitative, graphical, biostatisical, and probabilistic health information needed to make effective health decisions [48].

## Methods/design

### Study method

The planned systematic review will follow a comprehensive process and methodological guidelines suggested by other studies to synthesize the diverse forms of research evidence [6, 9, 24, 26]. Our review approach will largely be informed by conventional methods of conducting systematic reviews and will adhere to the PRISMA guidelines [30]. We will supplement our approach to accommodate the nature of the existing literature (for example, the impact of HL on chronic disease management) and different study designs (i.e. randomized controlled trials (RCTs), observational studies). The merit in including RCTs and observational studies is that the results of our review will reflect the rich and emerging literature base in this field as well as generate knowledge that could be applied in studies aiming to develop measurement tools for their studies.

### Literature search

**Fig. 1:**
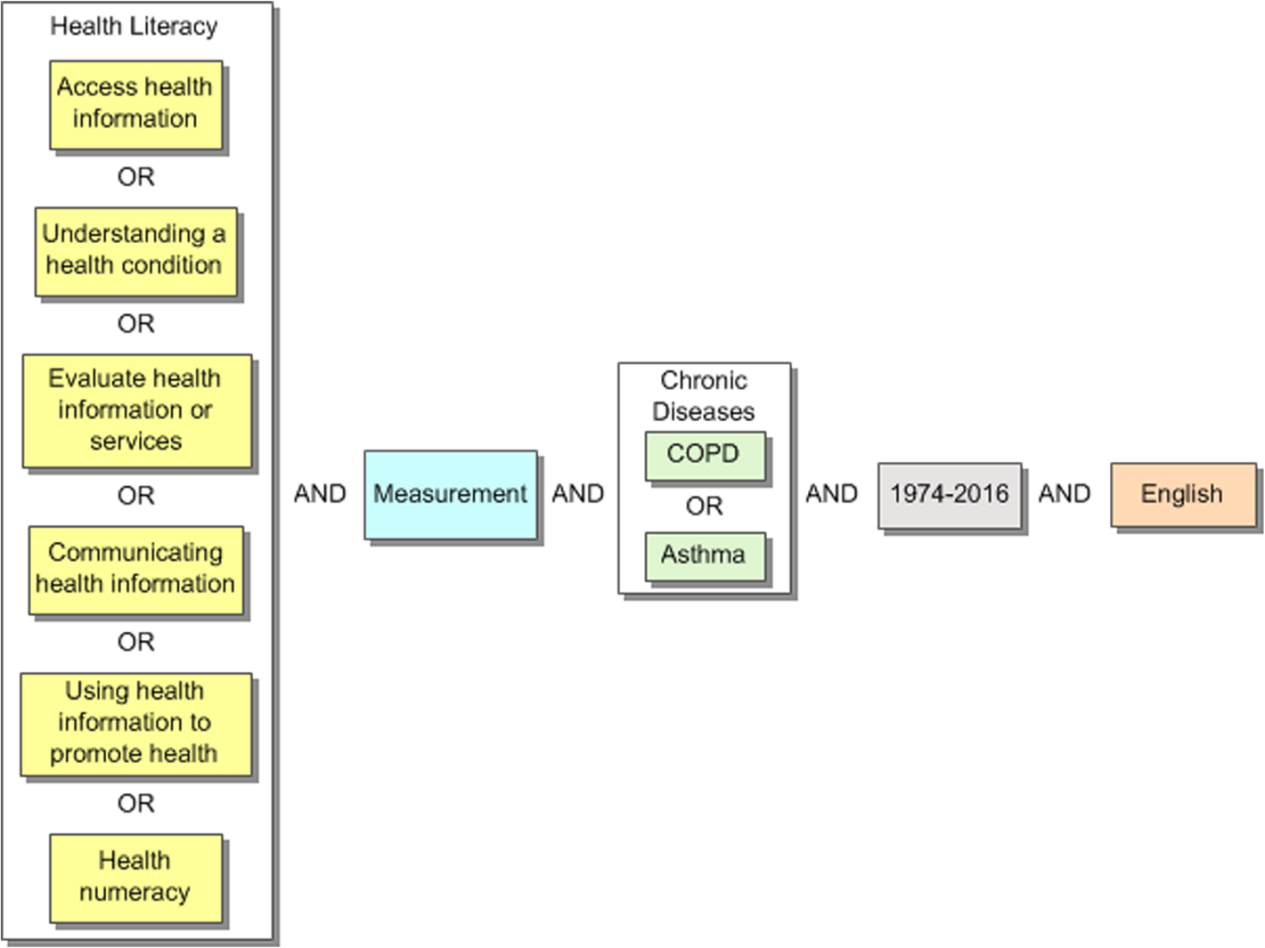
Health literacy search conceptualization

Our search will include the following concepts: (a) health literacy domains (access, understand, evaluate, communicate, and use) and health numeracy, (b) chronic diseases (asthma and COPD), and (c) measurement. The results will be limited to the years 1974 to 2016 and the English language (see Fig. 1 for search concepts). It is expected that this search will include papers pertaining to the development of measures, evaluation and critiques of the psychometric properties of measures, validation studies on measures, and reviews of measures. A template search strategy will be developed with these concepts and translated into the other databases (see Additional file 3).

“HL” as a recognized term came into use around 1974 but only became a MeSH term in MEDLINE in 2006; consequently, we will need to apply a broader approach to searching and look at each of the components of HL individually as they may not be included in the term “health literacy”. Measurement encompasses a variety of relevant terms. In MEDLINE, applicable MeSH terms may include “surveys and questionnaires”, “educational measurement”, “psychometrics”, and “health survey” to name a few. To ensure our searches are sensitive, we will include keywords to capture terms that may have been missed in our subject terms, for example, (assessment adj3 tool?).mp. We will also consider using a measurement search filter [39].

The published literature will be obtained through the University of British Columbia’s (UBC) library resources. The review will include database searches from the following disciplines: health (MEDLINE, Embase, CINAHL, and the Cochrane Central Register of Controlled Trials), sociology (Sociological Abstracts), anthropology (Anthropology Plus), education (ERIC), and general databases (Web of Science, Academic Search Complete), and topic-specific electronic databases from the fields of health, measurement, and education. A preliminary search will be developed in MEDLINE (Ovid) based on the studies in several review articles [6, 28]. The subject headings from the included papers will be examined, and appropriate MeSH headings and keywords will be used to construct the initial search.

The titles of papers that have been screened and meet the inclusion criteria will also be searched in the Social Sciences and Sciences Citation Indexes (Web of Science) and Elsevier ScienceDirect for citing articles, which may increase the yield of included articles. Reviews and the primary literature will be primarily examined first. Library catalogues will be searched for relevant books on measures, and searches will be performed for bibliographies of health literacy. The references from all selected papers will be reviewed. We will contact experts and search pertinent organizations’ websites to identify further publications not retrieved in the searches. The study team has established an advisory panel (AP) of experts and leaders in HL both nationally and internationally, and the names of relevant experts will also be searched (see Additional file 4).

Journals will be hand searched for possible papers, which will include the main journals known to have published HL papers such as *Health Promotion International*, *Health Affairs*, *Journal of Health Communication*, and *Annals of Internal Medicine*. Searches will be performed using several search engines using selected search terms from the strategy. Dissertations will be looked at from EThOS British Theses Group, Theses Canada Portal, and ProQuest Dissertations & Theses Global. Key conferences will be searched through the Internet looking for relevant proceedings, papers, and key authors including annual HL research conferences during the last 15 years.

During study selection, the names of measures meeting the inclusion criteria will become evident which will lead to new searches specific to those instruments. Most instruments do not have designated subject headings, so keyword searches will be critical to capture all variations of each measure’s name. It is expected that for many measures there will be a reasonable number of papers, so searching the instrument name will be sufficient. For some instruments, the name plus a measurement search filter [39] will capture relevant studies.

An experienced health research librarian (MD) with extensive knowledge in conducting systematic reviews will design and implement search strategies to identify evidence using the abovementioned databases. Previous work on systematic reviews completed by members of the research team (MD, JMF, and IP) will also inform our search strategies to identify and include in the current review. Search results will be imported into a reference management database (e.g. Refworks), and duplicates will be removed before review. Hand searching will be done by two reviewers (JS and IP) who will also conduct the final reviews. The total number of included studies at each stage of the systematic review will be recorded, and the results will be summarized in a PRISMA flow chart for the final report. A record of all project decisions will be recorded.

### Study selection

Two reviewers (JS and IP) will independently review and apply the eligibility criteria during study selection. These studies could include tool development, use, and validation studies. During the first stage, the titles and abstracts from the searches will be reviewed. If there is disagreement, a third reviewer (LN) will be asked to help resolve any disagreement between the two reviewers. Common agreement on selected articles will be considered for stage 2 of the full-text review. A third reviewer will resolve any disagreement on the inclusion of a study selected. The study selection process will be pilot tested with the two reviewers for both stages to ensure a high degree of inter-rater agreement. The selected studies after the stage 2 review will proceed to data extraction.

### Data extraction

Data extraction will be undertaken by two reviewers (JS and IP) for the reviewed literature using two standardized data extraction forms developed for this study (one for measurement tools and one for validation studies) (see Additional file 5). The data will be first extracted and entered into MS Excel spreadsheets in tabular form by JS and checked for accuracy and completeness by IP. Several papers will be pilot tested to ensure agreement and clarify decisions between the reviewers. The data extraction form for the measures will include the mapping of the items onto the five domains. Since the items of the tools may be grouped together and do not stipulate the domain they represent, two reviewers will independently review the items based on the definitions for the HL domains and assign the items to the appropriate domains. A third reviewer will resolve disagreements. Measurement properties will be captured and extracted using all the sections of the COnsensus-based Standards for the selection of health status Measurement INstruments (COSMIN) checklist (reliability, validity, responsiveness, and interpretability) from validation studies [40, 41]. We will also collect data on whether patients were involved in the validation stages. Relevant information from reviews of measures will also be added to this data extraction form. The data extraction form for the validation studies will include the following: *general information* such as author, title, year published, country of origin, and language; *study characteristics* including study design; *instrument details* including conceptual framework/model, target population (chronic lung disease), gender, age, purpose or use of instrument, number and type of categories, scale design, and scoring; *utility characteristics*, for example, time to complete measure (length), level of reading ability, strengths, weaknesses, and recommendations for measurement of HL; and *psychometric properties* such as internal structure (factor analysis), SEM, correlations with other variables, clinically important difference, and IRT-based analyses. Any other papers related to the measure will also be captured. We will contact the authors of relevant studies to obtain missing data.

### Quality assessment

An assessment tool will be developed to rate the level of evidence available on each measure. Key components will include a conceptual model or framework developed prior to items in the measure for one or more of the HL-included domains, a population that should be focused on asthma or COPD adults, and a detailed description of the development and initial validation process such as information on psychometric properties, additional studies validating the measure through RCTs, and/or observational studies and additional reviews or critiques regarding the measure and its measurement properties. The quality of validation studies will also be assessed using the appropriate quality assessment tools (e.g. risk of bias tool) [42, 43]. All sections of the COSMIN checklist [40, 41] will be used to evaluate papers that review the methodological components of an instrument. For quality assessment, a two-stage process will be used, in which two reviewers will work independently to complete the process of included articles. Disagreement will be resolved by discussion and if necessary adjudicated by a third reviewer. Pilot testing will also occur with the assessment tool to ensure clarity of components and rating levels.

### Synthesis and reporting

We will develop a final report that summarizes the methods of the review such as the criteria to identify and include studies and details of study designs. The report will conform to recommendations in the PRISMA checklist [30]. The formal analysis of the results will be a synthesis of the identified tools and selected studies to determine the distribution of the core domains and their subdomains in the development of a HL measurement tool to be used among asthma and COPD patients. A table of characteristics with variables from each tool and validation study will be included in the final report as well as a map of the structure of the core domains. The variables to be described in the synthesis include identifying the priorities of the domains, strengths and weaknesses of the tools, length of the tool (number of items in each domain and subdomains), time for completion, reading ability, format (scoring and scaling), and psychometric properties including reliability, validity, responsiveness, interpretability, internal structure (factor analysis), response processes, SEM, correlations with other variables, clinically important difference, and IRT-based analyses. Measures will be ranked based on key characteristics such as the underlying model or framework used to develop the measure, evidence of measurement properties, and validation studies that have incorporated the measure to test one or more of the domains with the asthma and COPD patient population.

## Discussion

Low HL has been associated with poor health outcomes in a variety of chronic conditions, including respiratory diseases (e.g. asthma and COPD). On the other hand, improved HL is associated with better health outcomes and lower costs. In addition, there is a recent recognition that chronic disease management, which is currently the preferred strategy for addressing the increasing rates of chronic diseases, can be improved through increasing the HL skills of patients as well as the communication skills of physicians and other health care professionals. A call to embrace the importance of HL in the context of chronic respiratory disease management has thus occurred in parallel with the increased awareness of the importance of comprehensively measuring HL due to the fact that existing measurement tools do not optimally help clinicians and researchers identify issues with access to health information or important gaps between understanding and evaluating health information and between communicating and using such information. We believe that this systematic review is timely and will make a valuable contribution to fill an existing research gap in the HL field. The findings of this review will be combined with the information collected from patient-engaged focus groups and key-stakeholder interviews enabling our team to develop a patient-centric and professional-perspective HL measurement tool for asthma and COPD management. The proposed tool will assist researchers and clinicians to not only measure patients’ HL in a comprehensive way but also to identify areas where work is needed to improve HL skills. Such a measurement tool will likely lead to the improvements of health outcomes and quality of life of patients with respiratory disease and has the potential to be adapted for other chronic conditions.

## Additional files

Additional file 1: PRISMA-P 2015 checklist. (DOCX 30 kb) Additional file 2: HL domain examples. (DOCX 31 kb) Additional file 3: Preliminary MEDLINE (Ovid) search. (DOCX 13 kb) Additional file 4: Relevant agencies. (DOCX 31 kb) Additional file 5: Health literacy data extraction fields for tools (draft). (DOCX 35 kb)

## Abbreviations

CCHL: Calgary Charter on Health Literacy
CEPHL: Canadian Expert Panel on Health Literacy
COPD: chronic obstructive pulmonary disease
COSMIN: COnsensus-based Standards for the selection of health status Measurement INstruments
HL: health literacy
NVS: Newest Vital Sign
PRISMA: Preferred Reporting Items for Systematic Reviews and Meta-Analyses
REALM: Rapid Estimate of Adult Literacy in Medicine
TOFHLA: Test of Functional Health Literacy in Adults
