# Supplementary material for: The application of health literacy measurement tools (collective or individual domains) in assessing chronic disease management: a systematic review protocol
Source: Syst Rev. 2016 Jun 7;5:97. doi: 10.1186/s13643-016-0267-8 (PMC4897812; doi:10.1186/s13643-016-0267-8)
Supplement: Additional file 2: — HL domain examples. (DOCX 31 kb) [file 13643_2016_267_MOESM2_ESM.docx]

**The application of health literacy measurement tools (collective or individual domains) in assessing chronic disease management: a systematic review protocol**

**Additional file 2**

**HL domain examples**

| **Access** | **Understand** | **Evaluate** | **Communicate** | **Use** |
| --- | --- | --- | --- | --- |
| I have the skills to ASK others for the health information that I want. | I have the skills to UNDERSTAND the health information that I find.  I have the skills to ASK others to clarify any health information that is unclear.  How often are appointment slips written in a way that is easy for you to read and understand?  How often are medical forms difficult for you to understand and fill out?  How often do you have difficulty understanding written information your health care provider gives you?  How often do you have problems learning about your medical condition because of difficulty understanding written information?  How confident are you filling out medical forms by yourself?  How often do you have someone help you read hospital materials?  How often are you unsure on how to take your medication(s) correctly because of problems understanding written instructions on the bottle label? | How you judgment or conclude about the health information presented to you by [doctor, hospital, …] to make decision to apply the information?  How you think the information presented to you is accurate, comprehensive and relevance?  I have the skills to MAKE SENSE of health information that is inconsistent  I have the skills to PICK OUT the health information that I want | I have the skills to SHARE with others the health information that I have learned | I am confident in applying the obtained information in my decision for my health |
