# Supplementary material for: The application of health literacy measurement tools (collective or individual domains) in assessing chronic disease management: a systematic review protocol
Source: Syst Rev. 2016 Jun 7;5:97. doi: 10.1186/s13643-016-0267-8 (PMC4897812; doi:10.1186/s13643-016-0267-8)
Supplement: Additional file 3: — Preliminary MEDLINE (Ovid) search. (DOCX 13 kb) [file 13643_2016_267_MOESM3_ESM.docx]

**The application of health literacy measurement tools (collective or individual domains) in assessing chronic disease management: a systematic review protocol**

**Additional file 3**

Preliminary MEDLINE (Ovid) Search

Database: Ovid MEDLINE(R) In-Process & Other Non-Indexed Citations and Ovid MEDLINE(R) <1946 to Present>

Search Strategy:

----------------------------------------------------------------

1 health literacy/ (2464)

2 health literacy.tw. (3573)

3 or/1-2 (4569)

4 Health/ (19325)

5 communication/ or access to information/ or communication barriers/ or health communication/ or information seeking behavior/ (78162)

6 information literacy/ (129)

7 literacy.tw. (10430)

8 ((access$ or seek$) adj5 information).tw. (15719)

9 (literac$ adj5 information).tw. (614)

10 or/5-9 (101597)

11 4 and 10 (807)

12 Health Knowledge, Attitudes, Practice/ (81606)

13 health education/ (54598)

14 consumer health information/ (2311)

15 patient education as topic/ (74445)

16 or/11-15 (197014)

17 Questionnaires/ (337065)

18 Educational Measurement/ (30218)

19 Psychometrics/ (59513)

20 (measures or measurement or test? or assessment or screen or screening or instrument).tw. (3003306)

21 or/17-20 (3245730)

22 16 and 21 (66008)

23 exp Asthma/ (112972)

24 lung diseases, obstructive/ or bronchitis/ or bronchiolitis/ or bronchitis, chronic/ or pulmonary disease, chronic obstructive/ or pulmonary emphysema/ (76726)

25 23 or 24 (180011)

26 16 and 21 and 25 (1528)

27 3 and 21 and 25 (55)

28 26 or 27 (1554)

29 limit 28 to yr="1985 -Current" (1526)

30 limit 29 to English language (1341)

31 comment/ or editorial/ or letter/ or news/ (1686865)

32 30 not 31 (1326)

33 limit 32 to "review articles" (117)

34 limit 32 to systematic reviews (70)

35 33 or 34 (150) [Reviews]

36 32 not 35 (1176)

**Note**

This search is NOT intended to be exhaustive.

The MeSH headings from the 11 papers found in the Jordan/Collin papers and the MeSH headings from the 3 reviews (see below) were used to help develop this preliminary search.

1. Collins SA, Currie LM, Bakken S, Vawdrey DK, Stone PW. Health literacy screening instruments for eHealth applications: a systematic review. J Biomed Inform. 2012 Jun;45(3):598-607.

2. Jordan JE, Osborne RH, Buchbinder R. Critical appraisal of health literacy indices revealed variable underlying constructs, narrow content and psychometric weaknesses. J Clin Epidemiol. 2011 Apr;64(4):366-79.

3. Berkman ND, Sheridan SL, Donahue KE, et al (2011). Low Health Literacy and Health Outcomes: An Updated Systematic Review. *Ann Intern Med*, 55:97-107
