# Supplementary material for: The application of health literacy measurement tools (collective or individual domains) in assessing chronic disease management: a systematic review protocol
Source: Syst Rev. 2016 Jun 7;5:97. doi: 10.1186/s13643-016-0267-8 (PMC4897812; doi:10.1186/s13643-016-0267-8)
Supplement: Additional file 4: — Relevant agencies. (DOCX 31 kb) [file 13643_2016_267_MOESM4_ESM.docx]

**The application of health literacy measurement tools (collective or individual domains) in assessing chronic disease management: a systematic review protocol**

**Additional file 4**

**Relevant agencies**

| **USA** | **Canada** | **Other** |
| --- | --- | --- |
| **Literacy organizations**   - Agency for Healthcare Research and Quality - American Medical Association - Canyon Ranch Institute - Centers for Disease Control and Prevention – Health Literacy Division - Harvard School of Public Health - Health Literacy Missouri - Institute of Medicine - Medical Library Association Health Information Literacy - RTI International   **Chronic diseases**   - American Asthma Foundation - COPD Foundation USA - American Thoracic Society - American Lung Association | **Literacy organizations**   - Canadian Public Health Association - DataAngel - Hamilton Health Science Centre - Health Quality Ontario - Public Health Agency of Canada - The Centre for Literacy   **Chronic diseases**   - Asthma Society of Canada - BC Lung Association - Canadian Lung Association - Ontario Lung Association - Asthma Foundation - COPD Canada | **Literacy organizations**   - World Health Organization (WHO) Publications   **Chronic diseases**   - Asthma Australia - Asthma Foundation New Zealand - British Thoracic Society |
