# Supplementary material for: The application of health literacy measurement tools (collective or individual domains) in assessing chronic disease management: a systematic review protocol
Source: Syst Rev. 2016 Jun 7;5:97. doi: 10.1186/s13643-016-0267-8 (PMC4897812; doi:10.1186/s13643-016-0267-8)
Supplement: Additional file 5: — Health literacy data extraction fields for tools (draft). (DOCX 35 kb) [file 13643_2016_267_MOESM5_ESM.docx]

**The application of health literacy measurement tools (collective or individual domains) in assessing chronic disease management: a systematic review protocol**

**Additional file 5**

**Health Literacy Data Extraction Fields for Tools (DRAFT)**

| Tool ID |  |
| --- | --- |
| Name of tool |  |
| Name of person extracting data |  |
| Year of publication |  |
| Where was it developed (country) |  |
| Who developed the tool |  |
| Purpose of the tool |  |
| Target population for which the tool was designed (gender, age, level of education, chronic disease) |  |
| Versions of the tool (e.g., short form, revised) |  |
| Version in language other than English |  |
| Who administers the tool (e.g., self-administered, interviewer-administered) |  |
| Total number of items |  |
| Sections (topics) in the tool |  |
| Number of items in Access domain |  |
| Number of items in Understand domain |  |
| Number of items in Evaluate domain |  |
| Number of items in Communicate domain |  |
| Number of items in Use domain |  |
| Number of numeracy items |  |
| Number of items not measuring health literacy |  |
| How is the tool scored |  |
| Strengths of the tool |  |
| Weaknesses of the tool |  |
| Date of completion of data extraction |  |
| Notes |  |

**Tool Items and their Domains (DRAFT)**

**(Completed by two reviewers independently if domains not stipulated)**

| Data Extractor | Tool ID | Item # | HL Domain | Notes |
| --- | --- | --- | --- | --- |
|  |  |  | Access |  |
|  |  |  |  |  |
|  |  |  | Understand |  |
|  |  |  |  |  |
|  |  |  | Evaluate |  |
|  |  |  |  |  |
|  |  |  | Communicate |  |
|  |  |  |  |  |
|  |  |  | Use |  |
|  |  |  |  |  |
|  |  |  | Numeracy |  |
|  |  |  |  |  |
|  |  |  | Not HL |  |
|  |  |  |  |  |

**Health Literacy Data Extraction Fields for Validation Studies (DRAFT)**

| Name of person extracting data |  |
| --- | --- |
| Tool ID |  |
| Authors |  |
| Title of paper |  |
| Year of publication |  |
| Published or grey literature? |  |
| Name of journal (if applicable) |  |
| Geographic location of development/validation |  |
| Is this paper a development and/or validation study? |  |
| Purpose of the paper/study (include hypotheses being tested) |  |
| Study design |  |
| How was the sample obtained? |  |
| Description of the sample (gender, age, level of education, chronic disease) |  |
| Were patients and/or caregivers asked to provide input on content/face validity? |  |
| Which version of the tool is being developed/validated |  |
| Underlying constructs of the tool |  |
| Reported time to participants to complete the tool |  |
| Readability - scale used and result |  |
| Strengths of the study |  |
| Weaknesses of the study |  |
| Internal structure (factor analysis) |  |
| Response processes |  |
| Standard error of measurement (SEM) |  |
| Correlations with other variables |  |
| Clinically important difference |  |
| Item response theory (IRT)-based analyses |  |
| Includes discussion of internal consistency (section from COSMIN) |  |
| Includes discussion of reliability (section from COSMIN) |  |
| Includes discussion of measurement error (section from COSMIN) |  |
| Includes discussion of content and face validity (section from COSMIN) |  |
| Includes discussion of structural validity (section from COSMIN) |  |
| Includes discussion of hypotheses testing (section from COSMIN) |  |
| Includes discussion of cross-cultural validity (section from COSMIN) |  |
| Includes discussion of criterion validity (section from COSMIN) |  |
| Includes discussion of responsiveness (section from COSMIN) |  |
| Includes discussion of interpretability (section from COSMIN) |  |
| Date of completion of data extraction |  |
| Notes |  |
